# 2473 Cardiac injury due to *Streptococcus pneumoniae* invasion during severe pneumococcal pneumonia in a novel nonhuman primate model

**DOI:** 10.1017/cts.2018.51

**Published:** 2018-11-21

**Authors:** Luis F. Reyes, Cecilia A. Hinojosa, Nilam J. Soni, Julio Noda, Vicki T. Winter, Melissa A. de la Garza, Jacqueline J. Coalson, Luis Giavedoni, Antonio Anzueto, Carlos J. Orihuela, Marcos I. Restrepo

**Affiliations:** 1 Division of Pulmonary Diseases & Critical Care Medicine, The University of Texas Health Science Center at San Antonio, San Antonio, TX, USA; 2 Division of Pulmonary Diseases & Critical Care Medicine, South Texas Veterans Health Care System, San Antonio, TX, USA; 3 Department of Microbiology, The University of Alabama at Birmingham, Birmingham, AL, USA; 4 Department of Pathology, The University of Texas Health Science Center at San Antonio, San Antonio, TX, USA; 5 Texas Biomedical Research Institute, San Antonio, TX, USA

## Abstract

OBJECTIVES/SPECIFIC AIMS: The aims of this study are (1) to develop and characterize a novel nonhuman primate model of pneumococcal pneumonia that mimics human disease; and (2) determine whether *Streptococcus pneumoniae* can: (a) translocate to the heart, (b) cause adverse cardiac events, (c) induce cardiomyocyte death, and (d) lead to scar formation during severe pneumonia in baboons. METHODS/STUDY POPULATION: Six adult baboons (*Papio cynocephalus*) were surgically tethered to a monitoring system to continuously assess their heart rate, temperature, and electrocardiogram (ECG). A baseline transthoracic echocardiogram, 12-lead ECG, serum troponin-I levels, brain natriuretic peptide, and heart-type fatty acid binding protein (HFABP) levels were obtained before infection and at the end of the experiment to determine cardiovascular damage during pneumococcal pneumonia. Animals were challenged with 108 colony-forming units of *S. pneumoniae* in the right middle lobe using flexible bronchoscopy. Three baboons were rescued with ampicillin therapy (80 mg/kg/d) after the development of pneumonia. Cardiac damage was confirmed by examination of tissue sections using immunohistochemistry as well as electron and fluorescence microscopy. Western-blots and tissue staining were used to determine the presence of necroptosis (RIP3 and pMLKL) and apoptosis (Caspase-3) in the cardiac tissue. Cytokine and chemokine levels in the heart tissue were determined using Luminex technology. RESULTS/ANTICIPATED RESULTS: Four males (57%) and three (43%) females were challenged. The median age of all baboons was 11 (IQR, 10-19) years old, which corresponds to a middle-aged human. Infected baboons consistently developed severe pneumonia. All animals developed systemic inflammatory response syndrome with tachycardia, tachypnea, fever, and leukocytosis. Infection was characterized by initial leukocytosis followed by severe leukopenia on day 3 postinoculation. Non-specific ischemic alterations by ECG (ST segment and T-wave flattering) and in the premortem echocardiogram were observed. The median (IQR) levels of troponin I and HFABP at the end of the experiment were 3550 ng/mL (1717–5383) and 916.9 ng/mL (520.8–1323), respectively. Severe cardiomyopathy was observed using TEM and H&E stains in animals with severe pneumonia. Necroptosis was detected in cardiomyocytes of infected animals by the presence of pMLKL and RIP3 in cardiac tissues. Signs of cardiac remodeling indicated by disorganized collagen deposition was present in rescued animals but not in the other animals. DISCUSSION/SIGNIFICANCE OF IMPACT: We confirmed that baboons experience cardiac injury during severe pneumococcal pneumonia that is characterized by myocardial invasion, activation of necroptosis, and tissue remodeling in animals rescued by antimicrobial therapy. Cardiac damage by invading pneumococci may explain why adverse cardiac events that occur during and after pneumococcal pneumonia in adult human patients.

